# Single-lesion paucibacillary leprosy: a retrospective study of 75 cases treated with the ROM scheme^⋆^

**DOI:** 10.1016/j.abd.2020.11.015

**Published:** 2022-01-15

**Authors:** Silmara Navarro Pennini, Josineide de Oliveira Novo França, Paula Frassinetti Bessa Rebello, Sinésio Talhari

**Affiliations:** a Tropical Medicine Program, Fundação de Medicina Tropical Dr. Heitor Vieira Dourado, Manaus, AM, Brazil; b Universidade do Estado do Amazonas, Manaus, AM, Brazil; c Fundação de Dermatologia Tropical e Venereologia Alfredo da Matta, Manaus, AM, Brazil; d Universidade Federal do Amazonas, Manaus, AM, Brazil; e Scientific Initiation Grant, Fundação de Dermatologia Tropical e Venereologia Alfredo da Matta in Collaboration with Fundação de Amparo à Pesquisa do Estado do Amazonas, Manaus, AM, Brazil

Dear Editor,

Leprosy, an infectious disease caused by *Mycobacterium leprae*, remains a public health problem in Brazil. In 2018, 28,660 new cases were recorded, corresponding to 93% of patients diagnosed in the American continent. In the same year, Brazil, India, and Indonesia contributed with 80% of the 208,619 new cases reported worldwide.^1^

For operational purposes, in 2009, the World Health Organization (WHO) recommended classifying leprosy according to the number of skin lesions: paucibacillary (PB) leprosy – one to five lesions; and multibacillary (MB) leprosy – more than five lesions.^2^

Multidrug therapy (MDT), recommended by the WHO in 1982, was decisive for the global reduction in the prevalence of leprosy. PB cases are treated for six months with rifampicin and dapsone; and MB cases undergo 12 months of treatment with the same drugs associated with clofazimine.^3^

Given the knowledge about the possibility of spontaneous cure of single skin lesions PB cases (SSL-PB), the WHO, in 1998, recommended studies with a single dose of Rifampicin - 600 mg, Ofloxacin - 400 mg and Minocycline - 100 mg, a drug combination called the ROM scheme.^4^

One of the first studies with the ROM scheme was carried out in India with 1,483 patients. The ROM scheme was compared with the MDT treatment for PB cases. In this randomized clinical trial, 1,381 patients completed the study, and, at the end of 18 months, 99.2% of the cases attained clinical cure. There was no difference regarding the cure rates between the patients allocated to the two regimens, either MDT or ROM.^5^

In Brazil, from October 1997 to November 1998, a multicenter study was carried out, designed as a prospective cohort, consisting of 259 patients with SSL-PB leprosy: 75 in the state of Amazonas, 59 in Goiás, 80 in Rio de Janeiro and 45 in Rondônia. All were treated with the ROM scheme and had a median follow-up of 31 months. In the follow-up, 216 (83.4%) cases were considered to be cured.^6^, ^7^

According to data from the world literature and the results of the Brazilian study, the Brazilian Ministry of Health (MH) in 2002 recommended the ROM scheme as an alternative treatment to be used in referral centers.^8^

In the latest leprosy control guide (MH, 2017), there is no reference to the ROM treatment and there are no explanations about its exclusion.^9^ On the other hand, in the 2020 recommendations for the treatment of leprosy, the WHO continues to recommend the ROM scheme.^10^

This investigation aimed to assess the clinical evolution of 75 patients in the state of Amazonas allocated to the Brazilian study, mainly in relation to the occurrence of events such as reactions, neuritis, and retreatment.

For this analysis, a search was carried out in the database of the Notifiable Diseases Information System (SINAN) and its medical records. Only one medical record was not found. It also aimed to rescue patients for dermatological examination through telephone contact. None were found.

During the investigation of the SINAM database, no information was found on the reentry of patients due to therapeutic failure.

The analysis of the medical records showed that 33 patients returned to the service: 19 with complaints not related to leprosy and 14 to have the disease reassessed. According to existing information, four patients showed signs of probable reactivation of the disease five to six years after treatment – ​​three cases reported reactivation or an increase in the size of the lesion, and one, classified as an indeterminate case, developed neuritis (Table 1). The medical records had no information about positive biopsy or bacilloscopy results. The dermatological examinations were not performed by the dermatologists involved in this research protocol. The criteria for therapeutic failure, after 20 years, were exclusively clinical and there was no definitive proof of treatment ineffectiveness.

**Table 1:** Causes of single lesion leprosy retreatment after being treated with the ROM scheme in the state of Amazonas.

Follow-up	Classification	Clinical manifestation	Month of occurrence	Treatment
Three years^a^	BT	Reversal Reaction / MB histopathology	16	MDT-MB
	BT	Neuritis (two nerves)	05	PRED/MDT-MB
	TT	Reversal reaction	07	MDT-PB
	BT	Reversal reaction	05	PRED/MDT-PB
	BT	Increase in the number of lesions	12	MDT-PB
	TT	Reversal reaction	13	PRED/MDT-PB
	TT	Reversal reaction	13	MDT-PB
	TT	Reversal reaction	13	MDT-PB
	TT	Therapeutic failure/Enlargement of the initial lesion	63	MDT-PB
Twenty years	TT	Therapeutic failure/Enlargement of the initial lesion	60	MDT-PB
	BT	Therapeutic failure/Enlargement of the initial lesion	72	MDT-PB
	I	Therapeutic failure/Neuritis (one nerve)	62	PRED/MDT-PB

a Source: Rebello PF. Single-lesion paucibacillary leprosy: perspectives for implementing the single-dose multidrug therapy (Master’s Degree dissertation). Goiânia, Universidade Federal de Goiás; 2001.

In conclusion, it is emphasized that the ROM scheme is recommended by the WHO and should be considered as an alternative scheme for the treatment of PB cases with a single lesion, especially in referral centers.

## Financial support

Fundação de Amparo à Pesquisa do Estado do Amazonas (FAPEAM) – scientific initiation grant.

## Authors’ contributions

Silmara Navarro Pennini: design and planning of the study; critical review of the literature; collection, analysis, and interpretation of data; drafting, review and approval of the final version of the manuscript.

Josineide de Oliveira Novo França: critical review of the literature; collection, analysis, and interpretation of data; drafting and review of the manuscript.

Paula Frassinetti Bessa Rebello: design and planning of the study; critical review of the literature; analysis and interpretation of data; drafting and critical review of the manuscript.

Sinésio Talhari: Critical review of the literature; analysis and interpretation of data; drafting, critical review of the manuscript and approval of the final version of the manuscript.

## Conflicts of interest

None declared.
